# RIPK1 and RIPK3 inhibitors: potential weapons against inflammation to treat diabetic complications

**DOI:** 10.3389/fimmu.2023.1274654

**Published:** 2023-10-26

**Authors:** Dan Ke, Zhen Zhang, Jieting Liu, Peijian Chen, Yucen Dai, Xinhai Sun, Yanhui Chu, Luxin Li

**Affiliations:** ^1^ College of Life Sciences, Mudanjiang Medical University, Mudanjiang, China; ^2^ Heilongjiang Key Laboratory of Tissue Damage and Repair, Mudanjiang Medical University, Mudanjiang, China; ^3^ School of First Clinical Medical College, Mudanjiang Medical University, Mudanjiang, China; ^4^ Department of Thoracic Surgery, Union Hospital, Fujian Medical University, Fuzhou, China

**Keywords:** diabetes, diabetic complications, inflammation, regulatory cell death, receptor interacting protein kinase

## Abstract

Diabetes mellitus is a metabolic disease that is characterized by chronic hyperglycemia due to a variety of etiological factors. Long-term metabolic stress induces harmful inflammation leading to chronic complications, mainly diabetic ophthalmopathy, diabetic cardiovascular complications and diabetic nephropathy. With diabetes complications being one of the leading causes of disability and death, the use of anti-inflammatories in combination therapy for diabetes is increasing. There has been increasing interest in targeting significant regulators of the inflammatory pathway, notably receptor-interacting serine/threonine-kinase-1 (RIPK1) and receptor-interacting serine/threonine-kinase-3 (RIPK3), as drug targets for managing inflammation in treating diabetes complications. In this review, we aim to provide an up-to-date summary of current research on the mechanism of action and drug development of RIPK1 and RIPK3, which are pivotal in chronic inflammation and immunity, in relation to diabetic complications which may be benefit for explicating the potential of selective RIPK1 and RIPK3 inhibitors as anti-inflammatory therapeutic agents for diabetic complications.

## 1 Introduction

Diabetes mellitus (DM) is characterized by metabolic dysregulation resulting from impaired insulin secretion and/or insulin resistance (1). The consequential increased insulin resistance and disrupted glucose metabolism lead to chronic inflammation (2). Epidemiological findings imply that patients with type 2 diabetes showcase higher levels of acute phase reaction products and inflammatory mediators within their bloodstream (3). It has long been objectively recognized in clinical medicine that salicylic acid-based anti-inflammatory drugs can lower blood glucose levels (4). Basic medical research has found that inflammatory cytokines may impair insulin signaling due to “cross-talk” (5). Indeed, all the research findings till now has shown that pancreatic islets of T2DM patients exhibit detectable tissue inflammation which is accompanied by increased expression of inflammatory factors and immune cell infiltration. These are hallmark features of chronic inflammation, which leads to gradual progression of pathological fibrosis (6–9). The chronic low-grade inflammatory state is considered a crucial mechanism in the advancement of diabetic complications, including diabetic ophthalmopathy, diabetic cardiovascular complications, and diabetic nephropathy.

NF-κB pathway, which is the activation of inflammasome and cell death is thought to regulate the inflammation responsible for diabetic complications (10–12). NF-κB is a crucial moderator of the innate immune system and is instrumental in maintaining the normal physiological functions of the body. In response to inflammation, the body triggers a natural immune response, whereby immune-responsive cells release inflammatory agents whose transcription depends on NF-κB activation (13, 14). Rupture of necrotic or apoptotic cells causes the release of molecules of Damage-Associated Molecular Pattern (DAMPs). These substances are released into the interstitial space of cells or the blood circulation after rupture or necrosis of autologous cells, and are recognized as danger signals by receptors. This recognition leads to stimulation of the immune system, which results in inflammation (15, 16). Inflammasomes are crucial centers of innate immunity that facilitate the secretion of pro-inflammatory cytokines and regulate the inflammatory response via interactions with other cells (17). Both RIPK1 and RIPK3 are closely linked to these regulatory processes and are considered a highly promising target. Inhibiting their kinase activities has shown efficacy in treating several animal models of human diseases (18–21). The promising potential of RIPK1 inhibitors in treating autoimmune diseases, inflammation, acute illnesses (such as severe novel coronavirus pneumonia sepsis), and a range of other conditions has led to great anticipation for their development (22, 23).

While it is increasingly acknowledged that diabetes is an autoinflammatory condition and its complications stem from a prolonged low-grade inflammatory state, the development of drugs targeting RIPK1 and RIPK3 to alleviate diabetic complications remains inadequate. Since overactivation of RIPK1 and RIPK3 can lead to harmful inflammatory responses and tissue damage, understanding the precise mechanisms of RIPK1 and RIPK3 regulation of inflammation and the development of selective inhibitors are of utmost importance for the treatment of diabetes complications (19, 24). Therefore, this review firstly examines the primary inflammatory mechanisms of diabetic complications and highlights the intricate and crucial functions of RIPK1 and RIPK3 in these mechanisms. Subsequently, this paper provides a concise summary of the latest research on the relationship between diabetic complications and RIPK1 and RIPK3 and additionally explores potential issues and drug development targets.

## 2 Structure and function of RIPK1 and RIPK3

Receptor-interacting protein kinases RIPK1 and RIPK3 are key protein kinases in the organism involved in a wide range of biological processes. Overexpression or aberrant function of RIPK1 and RIPK3 has been shown to correlate with the onset and progression of many diseases, ranging from inflammatory responses to neurodegenerative diseases and tumors.

Structure is the basis of a protein’s function, and different structural domains enabling different functions for RIPK1 and RIPK3. Three structural domains can be identified for RIPK1: the N-terminal kinase domain, the intermediate structural domain and the C-terminal death domain (DD) (25). The N-terminal kinase domain of RIPK1 plays a crucial role in RIPK1-dependent apoptosis and necroptosis, with its kinase activity being essential (26). Caspase-8, a crucial apoptotic protein, induces apoptosis through the cleavage of RIPK1 at D324 and the suppression of RIPK1 activation (25).

The intermediate structural domain of RIPK1 is involved in regulating NF-κB signaling (27, 28). RIPK1 can undergo ubiquitination on various residues within its structural domain, leading to the formation of complex I, which serves as a critical scaffold (29). Hai-Bing Zhang’s team discovered that the intermediate structural domain in RIPK1 holds a Lys377 polyubiquitination site, which is essential for building the NF-κB-activated IKK kinase complex (30). This complex can undergo ubiquitination by E3 ubiquitin ligases, including inhibitor of apoptosis 1 (cIAP1) or cIAP2 (31–33). The Lys377 residue facilitates transforming growth factor-β-activated kinase 1 (TAK1) activation by serving as a binding hub for downstream signals (e. g. NF-κB) through recruitment of the K63 ubiquitin-binding proteins TAK1-binding protein 2 (TAB2) or TAB3. This, in turn, promotes TAK1 activation (34, 35). Activated TAK1 mediates inhibitory phosphorylation of RIPK1 through activation of IκB kinase and MAPK. This leads to activation of the NF-κB pathway, promoting survival signaling (35, 36).

The C-terminal DD structural domain of RIPK1 heterodimerizes with the death structural domains of TNFR1, TRADD, and FADD. Additionally, it homodimerizes with itself, ultimately promoting the formation of death receptor signaling complex II. This leads to the activation of the N-terminal kinase domain and the promotion of necrotic apoptosis (29, 37, 38). DAMPs released through necrotic apoptosis establish feed-forward amplification loops via NF-κB-dependent production of inflammatory cytokines, thus further amplifying the inflammation and forming the cycle (39–41).

RIPK3 comprises an N-terminal structural domain of kinase and RHIM. The kinase activity of RIPK3 is linked with necroptotic apoptosis. Wang’s group demonstrated that RIPK3 undergoes autophosphorylation at residue S227 and binds to MLKL (42), forming a stabilizing complex that ultimately results in necrotic cell death through the creation of a cleavage pore in the plasma membrane. Another domain, the receptor-interacting protein kinase homotypic interaction motif (RHIM), is present in both RIPK1 and RIPK3 and serves a vital function. The RHIM enables interaction among RIPK1 and RIPK3 and facilitates transduction downstream signal for inflammation and cell death by allowing binding to various cellular junctions (43–45). For instance, as early as 2012 (46), research revealed that ZBP1 (Z-DNA binding protein 1; also known as DAI or DLM1) could trigger necrotic apoptosis via homotypic interaction between RHIM and RIPK3. As research progressed, the structure of ZBP1 was thoroughly detailed, revealing the presence of two RHIM structural domains; however, only one of the domains was found to be functional (47). The functional RHIM interacts with RIPK1 and RIPK3 to trigger the transduction of inflammatory signal. In this process, RIPK1 and RIPK3 act as scaffolds and are not associated with kinase activity or cell death (48). In both ZBP1 and TLR adaptors, RHIM is also the primary mode of TRIF signaling (26, 49). And following these receptors and associated adaptors, RIPK1 employs various mechanisms to regulate different modes (50), including activation of NF-κB in complex I bound to TNFR1, which is independent of kinase function, and maintaining balanced control of inappropriate activation of RIPK3 and caspase-8 (26, 50, 51). However, the full understanding of how RIPK1 balances the signaling of these various factors is still lacking. It is already known that the RHIM structural domain shared by RIPK1 and RIPK3 may serve as an integrator of the pro-death upstream signaling facilitated by TRIF and regulate the unnecessary activation of RIPK3 by ZBP1 (24, 25, 49).

The varied functions of RIPK1 and RIPK3 can be simply categorized into two roles: a scaffolding role and a kinase role. Interestingly, the scaffolding effect does not align completely with the kinase action of the process. In RIPK1, its scaffolding function controls pro-survival signaling and pro-inflammation, while the kinase function regulates cell death. RIPK3 exhibits similarity to RIPK1 in that the process of scaffolding is linked with pro-inflammatory signaling, while the state of kinase activity is linked with necrotic apoptosis. However, current research on RIPK1 inhibitors focuses primarily on its kinase activity, with even fewer investigations on RIPK3 inhibitors and drugs specifically targeting the role of the scaffold have yet to be developed. If using kinases like TAK1 and TBK1 as drug targets for the regulation of RIPK1 and RIPK3, it is important to consider their impact on RIPK1 and RIPK3. Therefore, further advancement of studies on the role of the RIPK1 and RIPK3 scaffolds is urgently needed. Coupled with the fact that the structure of RIPK lends itself to the development of pharmacologically specific small molecule inhibitors, more in-depth studies could help physicians tailor specific targeted inhibitors to the risk of progression of each patient, making precision medicine a reality.

## 3 Role of RIPK1 and RIPK3 in the regulation of inflammation

RIPK1 and RIPK3 play a significant role in the transduction of repetitive signal within the body’s inflammatory response and are instrumental in regulating and activating multiple signaling pathways. RIPK1 and RIPK3 activate inflammation-related pathways like NF-κB, leading to the production of inflammatory factors and responses (52–54). Furthermore, both RIPK1 and RIPK3 have been linked to the activation of the NLRP3 inflammasome and are also implicated in the production of the pro-inflammatory cytokine IL-1β (55, 56). Recent studies have shown that both RIPK1 and RIPK3 play a role in the regulation of cell death pathway selection and balance. Moreover, RIPK1 has been found to activate numerous cell death pathways, like apoptosis and necroptosis, in response to external stimuli (25, 57).

### 3.1 Role of RIPK1 and RIPK3 in NF-κB signaling pathway

NF-κB signaling pathway comprises receptor and receptor-proximal signaling interface proteins, IκB kinase complexes, IκB proteins, and NF-κB dimers (57). These proteins function as transcription factors that form dimers and regulate the expression of genes encoding acute phase response proteins, cytokines, immunomodulatory molecules, and cell adhesion molecules, among others (58–60). NF-κB is involved in various biological processes, including immune and inflammatory responses, as well as tumorigenesis, through the regulation of gene expression (61, 62). Numerous extracellular stimulatory signals, such as pro-inflammatory cytokines, physicochemical stimuli, LPS, and others, can activate the NF-κB signaling pathway (63, 64). Here we will use TNF-α, a classical pro-inflammatory cytokine, as an example.

Nutritional overload in peripheral metabolic tissues results in the production of TNF-α, which bind to TNFR1 and activate pre-assembled TNFR1 trimers, prompting the formation of complex I (65–69). The membrane complex I, also referred to as TNF receptor signaling complex (TNF-RSC), is composed of TNFR1 associated-death domain (TRADD), TNFR1 associated-factor 2 (TRAF2), RIPK1, cellular inhibitor of apoptosis proteins 1 (cIAP1), cIAP2, and linear ubiquitin chain assembly complex (LUBAC) (70). TRADD and RIPK1 are recruited to the intracellular death domain (DD) of activated TNFR1 through their respective DD structural domains to initiate formation of complex I (71, 72). In complex I, TRADD recruits the E3 ubiquitin ligase IAPs and then adds the K63 ubiquitin chain to RIPK1 (20).

**Figure 1:**
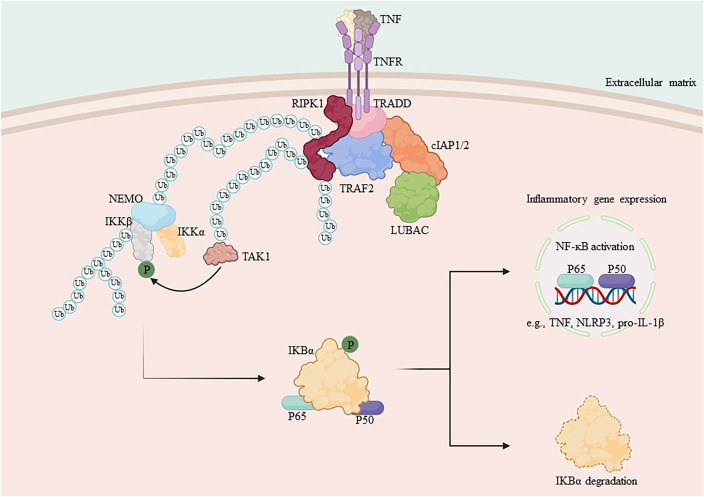
Mechanism of NF-κB activation by RIPK1. TNF-α binds to the membrane receptor TNFR1 and recruits TRADD, TRAF2, cIAP1/2 and RIPK1 to initiate the formation of complex I, which is composed of the ubiquitin chain assembly complex LUBAC and the E3 ubiquitin ligase. In complex I, LUBAC works with the E3 ubiquitin ligase to add the K63 ubiquitin chain to RIPK1, which binds to NEMO and recruits the IKK complex, activating the IκB protein and releasing NF-κB. Subsequent translocation of NF-κB to the nucleus promotes the transcription of pro-inflammatory genes and increases the expression of cytosolic inflammatory mediators.

RIPK1 serves as a crucial bridging protein in the classical NF-κB signaling pathway by enlisting the IKK complex, which comprises IKKα, IKKβ, and IKKγ, via its binding to IKKγ (also referred to as NEMO), an indispensable regulator of NF-κB signaling (31, 73, 74). Direct mediation of IKK complex activation occurs through an oligomerization- or ubiquitination-dependent mechanism of IKKγ (26, 75). The IKK complex, once activated, phosphorylates the IκB protein. As a result, phosphorylated IκB undergoes both ubiquitination and proteasomal degradation, thus releasing the NF-κB/Rel complexes (27, 76). The NF-κB/Rel complex is activated through post-translational modifications such as phosphorylation, acetylation, and glycosylation. Once activated, it is translocated into the nucleus and binds to target genes, thereby promoting transcription and increased expression of inflammatory mediators, including chemokines and cytokines (77–80) (**Figure 1**).

Appropriate responses from NF-κB are vital for the survival of cells, thereby promoting immunity by regulating the expression of genes associated with inflammation and neutralizing the cytotoxic effects of TNF-α (51, 81). During chronic diabetes, however, persistent hyperglycemia causes elevated blood levels of advanced glycation end products (AGEs). Subsequently, AGEs induce activation of IκB kinase (IKKβ), facilitate phosphorylation- and ubiquitination-mediated proteasomal degradation of the Iκβ protein inhibitor by binding with RAGE, and consequently prompt the release of NF-κB (82–84). The activated master transcription factor NF-κB moves to the nucleus and enhances the expression of different inflammatory cytokines (IL-1β, IL-6, TNF-α), which can lead to insulin resistance. This is a critical process where the ubiquitination scaffolding of RIPK1 plays a crucial role. RIPK1-deficient mice experience systemic multiorgan inflammation and typically die soon after birth (82, 85, 86).

Early studies on RIPK3, which has the same origin with RIPK1, concentrated on inducing cell death and activating NF-κB, but the findings were perplexing, for RIPK3 hindered the activation of NF-κB by both Toll-like Receptor 3 (TLR3) and the TLR4 signaling adapters TRIF and TNFR1 (86, 87). However, other studies have shown increased NF-κB activation after RIPK3 enhancement, leading to speculation that RIPK3 may not significantly contribute to NF-κB activation (88). However, subsequent research indicated that phosphorylation and degradation of IκBα, induced by tumor necrosis factor, TLR2, and TLR4, remained unaltered in cells with double knockout of RIPK3 (88–91). The expression of tumor necrosis factor, IL-6, and IL-1β induced by endotoxin decreased in double knockout RIPK3 mice. Moreover, LPS-induced phosphorylation and degradation of IκBα remained normal in double knockout RIPK3 bone marrow-derived macrophages (BMDMs) (92). However, the RelB-p50 heterodimer’s nuclear translocation was significantly hindered, while that of other NF-κB subunits remained unaffected (93). The findings indicate that although RIPK1 promotes the early phosphorylation and degradation of IκBα, RIPK3 controls the activation of NF-κB in a cell type-dependent manner downstream of IκBκα. Thus, while RIPK1 and RIPK3 frequently work together to induce cell death, they regulate the activation of NF-κB separately through different mechanisms. Additional all-encompassing proteomic analyses investigating the molecular interactions linked to RIPK1 and RIPK3 are necessary to unveil further mechanistic details of these regulations.

Furthermore, prior research has demonstrated that signal transduction of RAGE/NF-κB additionally triggers the development of NLRP3 inflammasome. In human monocytes, lipopolysaccharide signaling through TRIF activates the NLRP3 inflammasome, which recruits RIPK1, FADD, and caspase-8 to trigger K^+^-non-efflux-dependent NLRP3 activation (18, 94). Notably, in mice bone marrow stromal cells, TLR4 activation alone directly stimulates NLRP3 inflammasome activity mediated by RIPK1, RIPK3 and caspase-8 and is independent of cell death (95, 96). NLRP3 inflammasome respond to cellular stress signals and participate in the maturation and secretion of the critical cytokines, IL-1β and IL-18, leading to inflammation (97–100). Given the complex regulatory mechanisms involved, it would be reasonable to take the view that inhibiting the kinases RIPK1 and RIPK3 could modulate NLRP3 inflammasome levels through the cell death pathway. Necrotic apoptosis releases DAMPs, which activate NLRP3, leading to intracellular potassium loss. This, in turn, results in NLRP3 cleavage, activating caspase-1 and IL-1β and triggers a complex network of cellular responses that cause local and systemic inflammation (9). The latest studies indicate that intravenous immunoglobulin (IVIg) is capable of regulating the expression and activation of NLRP3 inflammasomes by reducing RIPK1 levels. Therefore, the inhibition of RIPK1 can help to reduce inflammation by regulating inflammasomes. However, due to the complexity and variety of cell types found in the human body, further experiments are necessary to confirm the utility of RIPK inhibitors.

### 3.2 Role of RIPK1 and RIPK3 in cell death

Cell death runs through the life activities of multicellular organisms, and countless diseases of living organisms arise from abnormal cell death, whether in excessive or inadequate amounts (101, 102). Regulatory cell death is a multifaceted process. With the development of the research, various modes of regulatory cell death have been identified, mainly categorized as apoptosis and necrotic apoptosis based on their effects. As previously mentioned, complex I activates NF-κB and supports cell survival. However, its short lifespan quickly transforms into the transition of the secondary cytoplasmic complex II, which regulates cell death. The different forms of complex II direct the cell towards different pathways of death (29, 37, 38).

Apoptosis is the-earliest-discovered regulated form of cell death that can be triggered by TNF-α through an exogenous pathway or by mitochondrial effectors via an endogenous pathway (103, 104). Caspases are crucial proteins that drive apoptosis. Complex IIa is the cytoplasmic complex consisting of FADD and caspase-8. The activated caspase-8 in complex IIa initiates the apoptotic program via a cascade reaction and cleaves necrotic mediators, including RIPK1 and RIPK3, among others (18, 25, 29). Caspase-8 cleavage of RIPK1’s C-terminal agonist domain at residue D324 is a crucial mechanism for inducing apoptosis and preventing both RIPK1 activation and necrotic apoptosis (105–107).

**Figure 2:**
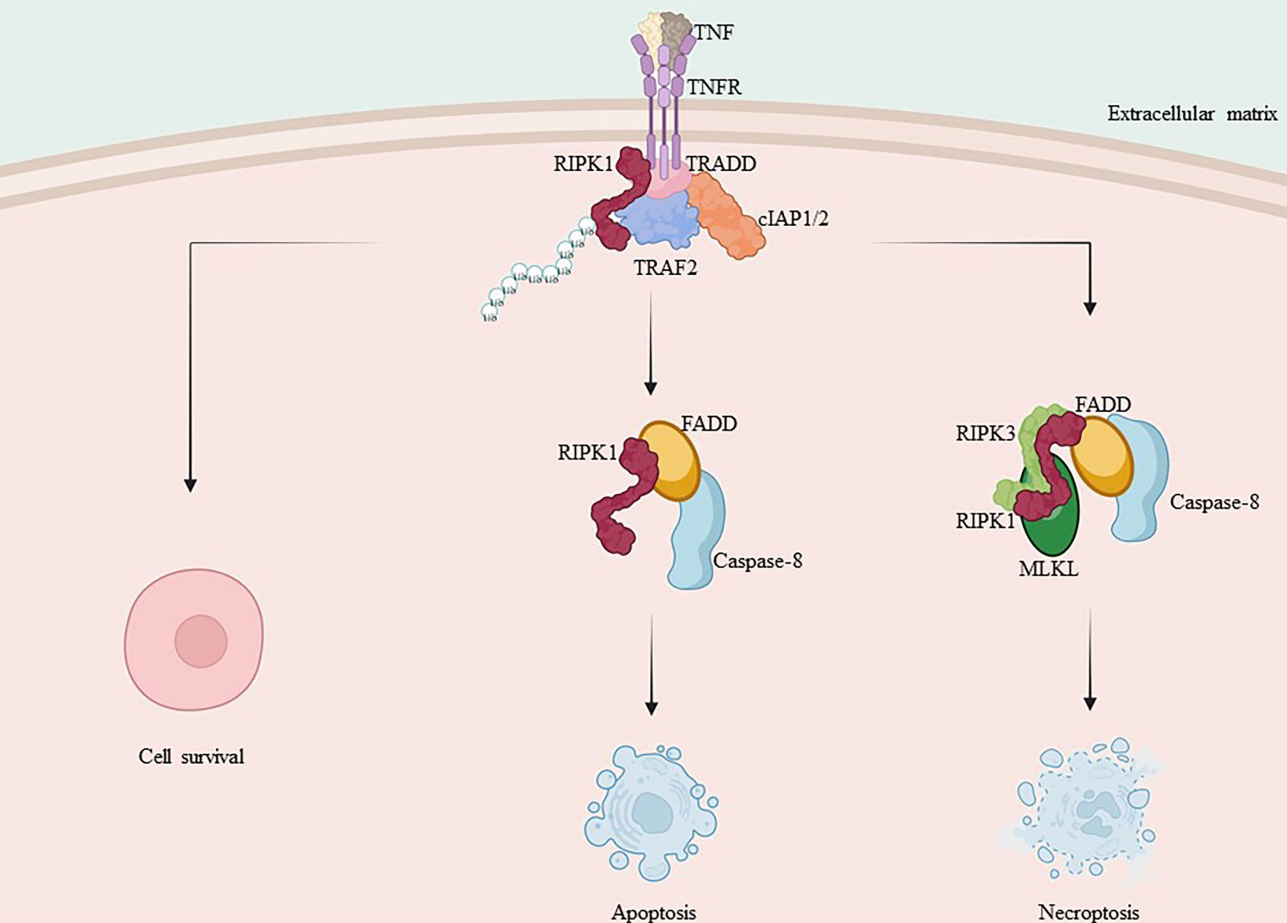
Mechanism of RIPK1 and RIPK3 in apoptosis and necroptosis. TNF-α binds to the membrane receptor TNFR1, recruiting TRADD, TRAF2, cIAP1/2 and RIPK1, initiating the formation of pro-survival complex I. Caspase-8 is activated and RIPK1 and TRADD in complex I bind to FADD, forming complex II a with activated caspase-8, promoting apoptosis. When caspase-8 activation is inhibited, RIPK1 and RIPK3 interact via RHIM isoforms, recruiting MLKL and forming complex II b, leading to necroptosis.

When caspase-8 activation is inhibited, RIPK1 is able to dimerize through its C-terminal DD, ultimately resulting in the activation of RIPK1 as well as the formation of complex IIb. This complex includes FADD, caspase-8, RIPK1, RIPK3, and MLKL (18, 29, 102). Subsequent ubiquitination of RIPK1 within complex IIb facilitates the activation of RIPK3 and MLKL. Additionally, RIPK3, which shares the same RHIM Domain as RIPK1, forms a necrosome with RIPK1 via a homotypic interaction, also referred to as complex IIc (18, 29). RIPK3 oligomers recruit MLKL to necrosomes, leading to its oligomerization (108). Oligomerized MLKL translocate to the plasma membrane through the Golgi-Microtubule-Actin pathway (109). and forms clusters of pores with tight junction proteins or modulates ion channel flux to induce necrotic apoptosis (**Figure 2**).

It should be noted that while RIPK1 in most cases is generally accepted as the upstream kinase of RIPK3, some research has demonstrated that RIPK3 can trigger necrotic apoptosis without reliance on RIPK1. Furthermore, this effect has been observed in cases where there is an overexpression of RIPK3 (15, 110–112).

Necrotic apoptosis is generally considered as a significant contributor to cell death and inflammation in numerous pathological contexts (112–115). For example, the important role of cell death in driving inflammation is provided by mutation of the proline-serine-threonine phosphatase-interacting protein 2 gene in mice (Pstpip2cmo), which causes inflammatory lesions in the bones and various degrees of skin and paw inflammation that closely resemble chronic recurrent multifocal osteomyelitis in humans (116). Necrotic and apoptotic cells display enlarged and swollen organelles along with early plasma membrane damage, leading eventually to rupture (115). The rapid loss of integrity of the plasma membrane leads to the release of DAMPs, which include IL-1α, HMGB1, and uric acid (117, 118). Since this type of inflammation is not associated with pathogen infection, it is known as aseptic inflammation. Like pathogen-induced inflammation, danger-associated molecular patterns (DAMPs) have the ability to activate both immune cells (such as neutrophils, macrophages, and dendritic cells) and nonimmune cells (like endothelial cells and fibroblasts) (119). These activated cells release numerous cytokines and chemokines, resulting in the recruitment of inflammatory cells and the activation of an adaptive immune response (120).

### 3.3 Role of RIPK1 and RIPK3 in inflammatory crosstalk

Although we have separately discussed regulatory cell death and the NF-κB pathway, but in fact these two processes they often work together. Infection, injury, and stress can lead to cytokine production to prime response by directly inducing regulatory cell death and triggering immune cell activation, leading to cytokine production (121). Dead cells initiate adaptive immunity by providing antigenic and inflammatory stimuli to dendritic cells (DCs). The DCs then activate CD8 T cells through a process called antigenic cross-stimulation (121).

However, Malek’s team discovered that the release of inflammatory mediators, including DAMPs, from dead cells was inadequate for initiating CD8 T cell crossover, and that successful crossover initiation required RIPK1 signaling and NF-κB to induce transcription within the dead cells (122). To orchestrate the adaptive immune response, inflammatory and cell death signaling pathways work together (123, 124). Receptors that elicit necrotic apoptosis also effectively induce NF-κB (51, 125), The effector molecule RIPK1 has been identified as a crucial mediator in regulating cellular activity and inflammation through the formation of common modules, complex I and complex II.

**Figure 3:**
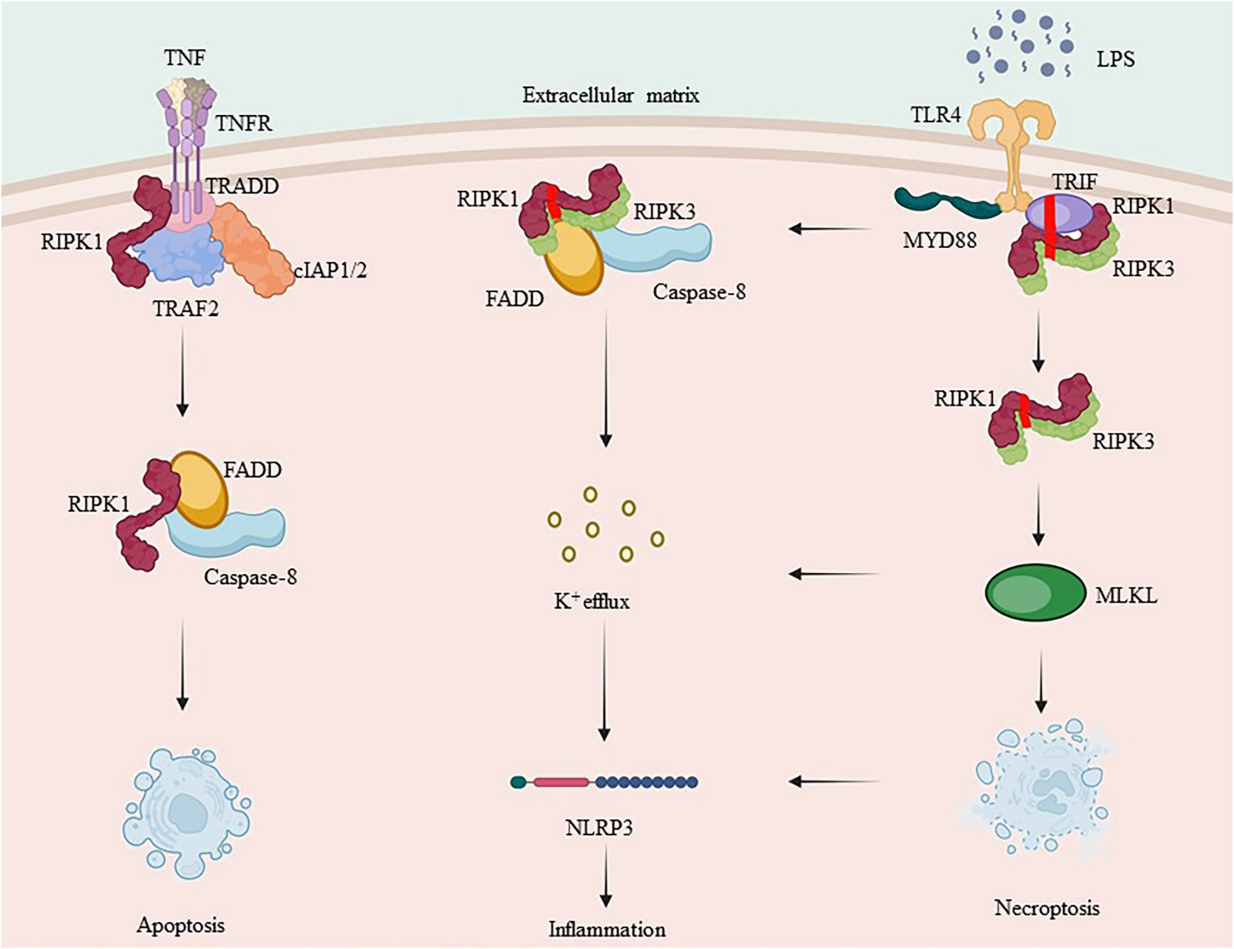
RIPK1 and RIPK3’s complex crosstalk in inflammation. TNF-α binds to the membrane receptor TNFR1 and recruits TRADD, TRAF2, cIAP1/2 and RIPK1 to initiate complex I formation. RIPK1, which is involved in complex I formation, is a key protein in both complex IIa and complex IIb. Ubiquitination of RIPK1 plays an essential role in NF-κB signaling. Caspase-8, when activated, forms complex IIa with RIPK1, leading to apoptosis, whereas inhibition of caspase-8 promotes necrotic apoptosis by forming complex IIb. RIPK3 can also independently induce the formation of inflammasome.

Overall, complex I promotes survival, complex IIa triggers apoptosis, and complex IIb induces necrosis. The type of complex II (either a or b) and caspase-8 status (either activated or inactivated) determine the mode of cell death, which can either be apoptosis or necrotic apoptosis. As a shared constituent of individual complexes, RIPK1 integrates different signals from external sources, including cytokines, growth factors, and pathogens, and regulates cellular behavior. Inflammatory cytokine production involves signaling through the TNF receptor (TNFR) superfamily and innate immune signal transduction through pattern recognition receptors (PRRs). Depending on the stimulus and environment, IAPs may ubiquitinate RIPK1, leading to inflammatory signal transduction via NF-κB, inhibiting RIPK3 necrosis-inducing complex formation, or inhibiting RIPK1/inflammatory pathways triggered by RIPK3 complex activation (57, 126, 127). Simultaneously activated immune cells produce cytokines that stimulate inflammation by activating pro-inflammatory genes. Additionally, they initiate regulated cell death, which closes a loop and amplifies the inflammatory response (**Figure 3**).

### 3.4 Progress in RIPK inhibitor development

As far back as 1876, researchers discovered that anti-inflammatory treatments provided benefits for diabetes, including the improvement of blood sugar in diabetic patients with the use of sodium salicylate (128). More than a century later, Shoelson and colleagues demonstrated that inhibition of the NF-κB pathway mediates this antidiabetic effect (129). NF-κB functions as a primary transcription factor in the upregulation of RAGE, inflammatory cytokines, and associated insulin resistance genes, and inhibition of NF-κB may be an effective treatment for diabetic complications (82). Blocking pro-inflammatory cytokines has been employed to treat other cytokine storm-related diseases, though with varied success. This underscores the limited comprehension of the underlying mechanisms (130). Currently, the primary objective of inflammation-targeted therapy against NF-κB is to hinder its activity. This can be achieved by inhibiting the phosphorylation of the IκBα protein to prevent its ubiquitination and degradation, or by hindering the DNA-binding activity of NF-κB (130). Similar to the mixed success of drugs blocking proinflammatory cytokines like TNF-α (131). Considering that NF-κB plays a crucial role in mediating normal immune response, inhibiting its function comes with the risk of inhibiting immune response while also reducing inflammation, how to balance this relationship is a major challenge in drug development. Thus, targeting the development of specific small molecules meticulously is necessary to ensure the vision of effective therapy.

Recent researches indicate that the upstream activating proteins of NF-κB, namely RIPK1 and RIPK3, have significant roles in immunity and inflammation. RIPK inhibitors have exhibited anti-inflammatory potential by obstructing inflammatory signal transduction. Developing highly selective inhibitors that target RIPK1 may offer innovative solutions for treating a broad range of diseases, including neurological disorders, inflammatory diseases, tumors, and sepsis (132–136). Therefore, a combination of inhibitors targeting RIPK1 and RIPK3 and hypoglycemic drugs may increase effectiveness in treating diabetes mellitus (137–139).

It has been proved in experiments that Nec-1, Nec-1s, and GSK872 are inhibitors that target RIPK1 and RIPK3 for treating inflammatory diseases, such as inflammatory response syndrome, diabetic ophthalmopathy, atherosclerosis, pancreatitis, nonalcoholic fatty liver disease, and ischemia-reperfusion injury of the brain, heart, and kidneys. These essential proof-of-concept studies have steered the development of RIPKs in pharmaceutical companies towards antagonists for clinical trials. Harris et al. disclosed in early 2016 that a superb benzoxazinone-like lead compound, which inhibits RIPK1, was discovered in a DNA-encoded compound library (GSK481) (140). After one year, they published another article on the optimization of GSK481 for acquiring a clinical candidate compound (GSK2982772) and presented its outstanding features (141). GSK2982772 specifically binds to RIPK1 and efficiently blocks TNF-dependent cellular pathways, including NF-κB and necrotic apoptosis-inducing inflammatory pathways. As a new anti-inflammatory compound, it reduces the spontaneous production of cytokines in ulcerative colitis tissues. Due to its favorable physicochemical properties and ADMET, it is anticipated to be a low-dose oral drug. The compound is undergoing Phase II clinical trials for the treatment of plaque psoriasis, rheumatoid arthritis, and ulcerative colitis. Phase I clinical trials for inflammatory bowel disease have been completed. Pharmaceutical companies, including GlaxoSmithKline, Sanofi, and Denali, are currently prioritizing the development of drugs that inhibit RIPK1. **Table 1** presents the pertinent developments in this area.

**Table 1:** Ongoing and completed clinical trials for RIPK inhibitors.

Compound	Target	Phase	Disease	Result	Clinical trial identifier
GSK2982772	RIPK1	1	Healthy adult	Sixty-five healthy subjects were enrolled. Two serious adverse events of 60mg b.i.d. were herpes zoster. Common drug-related adverse events were nasopharyngitis and headache.	NCT03590613(complete)
	RIPK1	2	Ulcerative colitis, UC	Twelve patients. 60mg was administered twice daily. Adverse events were mild, mainly headache.	NCT02903966(complete)
	RIPK1	1	Inflammatory bowel disease, IBD	Seventy-nine patients. Adverse events of moderate intensity, the most common adverse events were contact dermatitis and headache	NCT02302404(complete)
	RIPK1	1	Autoimmune disease, AID	Forty-five subjects.	NCT03266172(complete)
	RIPK1	2	Rheumatoid arthritis, RA	Fifty-two patients were enrolled. Adverse events were mild to moderate, related to arthralgia, headache, and peripheral swelling. One treatment-related adverse event was severe visual impairment and retinal vein thrombosis	NCT02858492(complete)
	RIPK1	2	Psoriasis	Sixty-five patients were enrolled. Two adverse events were verified in the 60mg bid and 60mg tid.bid groups	NCT02776033(complete)
SAR443122	RIPK1	2	Systemic lupus erythematosus, SLE	*	NCT04781816
	RIPK1	1	Corona Virus Disease 2019, COVID-19	C-reactive protein level in 68 hospitalized adult patients with severe coronavirus disease 2019	NCT04469621(complete)
SAR443820	RIPK1	1	Healthy Chinese and Japanese	*	NCT04982991(complete)
SAR443060	RIPK1	1	Amyotrophic lateral sclerosis, ALS	Fifteen patients with ALS. Adverse events were well tolerated. The most common emergent adverse events were medical device site irritation and catheter site related reactions.	NCT03757351
	RIPK1	1	Alzheimer’s disease, AD	Sixteen patients were enrolled. Adverse events were well tolerated, and the most common TEATs were confusion, headache, and procedural pain.	NCT03757325(complete)

Search ClinicalTrials.gov and the Clinical Trials Register for a list of all identifiable completed or ongoing clinical trials.

In conclusion, NF-κB and its associated kinases RIPK1 and RIPK3 hold potential as specific targets for chronic immune-related illnesses. However, due to the multifaceted role of NF-κB, targeting it may result in unintended repercussions, ultimately leading to a high incidence of side effects. Instead, utilizing the more nuanced RIPK1 inhibitors may be a better approach to targeting inflammation-related signals. Additionally, RIPK1, comprised of multiple structural domains (including the RHIM structural domain, DD structural domain, and kinase activity domain), coordinates cell death and innate immunity signals to orchestrate adaptive immunity. Currently, there are no effective inhibitors of RIPK3 in clinical trials. However, as noted previously, RIPK3 functions independently of RIPK1. Therefore, targeting other elements, such as specific downstream effector RIPK3, in the development of RIPK1 inhibitor medications could potentially enhance the drug’s therapeutic effectiveness in certain cases. Currently, the kinase activity of RIPK3 remains poorly understood, and development of drugs targeting RIPK1 holds more promise.

The development of RIPK1 inhibitors has primarily targeted its kinase active site, including the ATP binding site and hydrophobic pocket, with limited attention given to its scaffolding role. As a result, exploring and targeting scaffolding proteins at the intersection of cell death and host defense pathways may present novel therapeutic avenues for immunotherapy research.

## 4 Role of RIPK1 and RIPK3 in diabetic complications

The development of diabetic complications, including ophthalmopathy, cardiovascular complications, and nephropathy, is closely linked to the chronic low-grade inflammatory state caused by diabetes mellitus (142–144). Chronic hyperglycemia and insulin resistance result in the release of inflammatory catalysts and activation of immune cells, subsequently contributing to the onset and sustainability of the inflammatory response (145). Controlling the inflammatory response is crucial for preventing and treating diabetes-associated inflammatory complications.

### 4.1 Role of RIPK1 and RIPK3 in diabetic retinopathy

Diabetic retinopathy (DR) is a specific microvascular complication of diabetes mellitus that occurs in about one third of patients with DM, and with the incidence of DM increasing every year, DR has become a major cause of vision loss in the general population, along with blindness in patients with DM (146).

Microglia are resident macrophages in the retina that can monitor their surroundings and maintain tissue homeostasis by dynamically changing their morphology and function in response to changing cellular microenvironment (147). In DR, microglia proliferate and migrate from the inside of retina to the outside, secreting a variety of inflammatory cytokines, leading to increased vascular permeability, and causing intraretinal fluid accumulation and disruption of the blood-retinal barrier (147). Indeed, elevated chemokines and cytokines can be detected in the serum, vitreous, retina and atrial fluid of patients with DR, and inhibition of these inflammation-associated factors may be beneficial in halting angiogenesis and neurodegeneration in DR (148).

Upon recognition of hyperglycemia-generated DAMPs, microglia recruit RIPK1 as a cell death complex (26). RIPK1 mediates both its own activation and necroptosis. TNF-α released by activated microglia further activates the TNF-α-RIPK1-NF-κB signaling pathway to promote its transcription, leading to increased expression of IL-6 and IL-8 and activation of caspase-1 (149). In diabetic mouse model, activated caspase-1 promotes neovascularization with new vessels sprouting from the optic nerve head and small veins in the mid-peripheral retina (150).These vessels eventually bleed into the vitreous cavity due to fibrotic/glial cell vitreoretinal adhesion and traction, ultimately leading to retinal detachment, making RIPK1 a very important source target (151). In addition, in the vitro studies have shown that endothelial cell apoptosis and necroptosis increase in response to inflammatory cytokine stimulation, further leading to the release of DAMPs and promoting the inflammatory cycle (18, 152, 153). Necroptosis is cell death dependent on the activity of RIPK1 and RIPK3 kinases, and inhibition of RIPK1 and RIPK3 effectively reduces necroptosis. Inhibition of RIPK1 and RIPK3 affects the developmental progression of retinopathy in animal models of diabetes. In addition, Kate E. Lawlor et al. proved that RIPK3 directly regulates NLRP3 inflammasome activation in macrophages, but the exact mechanism involved is still unclear and further studies are needed to explore the possibility of RIPK3 as a therapeutic target for DR (154).

Inhibition of pro-inflammatory molecules has been confirmed to attenuate diabetes-induced vascular and neurodegenerative disease in animal models of DR (155), and studies using anti-inflammatory agents such as salicylates or minocycline in patients with DR have further demonstrated that modulation of the inflammatory response may be beneficial in preventing long-term irreversible vascular and neuronal damage (156–158). Rosenbaum et al. demonstrated that Nec-1 treatment prevents retinal cell death in a rat model of retinal ischemia, suggesting that RIPK1 is a promising target for DR (159). However, we have discussed that inflammation responds differently in different immune cells, so more research is needed to determine the precise molecular mechanisms and functions of each type of inflammatory cell in the pathogenesis and development of DR. It may pave the way for targeted drugs.

### 4.2 Role of RIPK1 and RIPK3 in diabetic cardiovascular disease

It is widely accepted that hyperglycemia is not inherently frightening for patients with diabetes; rather, what causes concern is the multitude of morbidity and mortality complications associated with the disease. Among these complications, cardiovascular complications, to which atherosclerosis (AS) is identified as the primary pathological mechanism contributing, are the primary cause of death in diabetic patients (160, 161).

AS initiates with inflammatory effects on endothelial cells that uphold the integrity and regular function of the arterial wall. The elevated NF-κB pathway stimulates endothelial cells, which leads to endothelial cell activation by inflammatory factors, such as TNF-α. When the fine balance of endothelial cells is compromised, monocytes adhere, and foam cells accumulate lipids. Low-density lipoprotein (LDL) deposition oxidation is closely linked to AS. Oxidized-LDL deposited in the endothelium induces RIPK3 expression in macrophages, promotes the phosphorylation of both RIPK3 and MLKL, and causes necrotic apoptosis in macrophages (108, 115, 162, 163). while the death of macrophages significantly affects the formation of necrotic cores and destabilization of plaques in advanced AS lesions (164, 165).

The necrotic core of the plaque is associated not only with macrophages but also with vascular smooth muscle cells. These cells play a role in the formation of vascular plaques during atherosclerotic lesions. Adhesion of these cells to the subendothelial space is associated with various cytokines. It is well known that neutrophils in diabetic patients secrete higher levels of cytokines and growth factors, such as TNF-α, IL-1β, and IL-8, than in healthy individuals (166, 167). The harmful and inflammatory impacts of TNF-α are primarily executed by controlling the crucial transcription factor NF-κB. Endothelial cells, triggered by TNF-α, display elevated RIPK1 levels to promote inflammation that relies on NF-κB (168–170).

Inflammasome also contributes to initiating this process. For instance, TNF-α can stimulate the synthesis of inflammasome, including the pivotal NLRP3, by upregulating NLRP3 expression in human vascular smooth muscle cells (171). Additionally enhancing neutrophil migration to the inflammation site, phagocytosis, cytosolic protease release, reactive oxygen species (ROS) production, and apoptosis are associated with increased NF-κB activation and inflammatory cytokine production due to ROS overproduction. Furthermore, inhibiting NF-κB may help mitigate endocardial inflammation as well as the reorganization of cell-substrate interactions (168–170). Indeed, NF-κB has a direct impact on cell death, calcium management, cell attachment, and the production of inflammatory proteins such as COX2 and iNOS. Suppressing NF-κB prevents hypertrophy and heart failure in a mouse model of pressure overload as well as after coronary artery ligation (27, 172). Furthermore, RIPK3 has the capability to produce mature IL-1β through its activation of the NLRP3 inflammasome, subsequently leading to the generation of an inflammatory response (173).

The formation and expansion of necrotic cores within plaques are significant factors in the pathology of unstable atherosclerotic plaques (174, 175). Heng Chen et al.’s team demonstrated that Arctiin protects the rat heart by reducing necrotic apoptosis through scavenging ROS and restoring mitochondrial functions (176). Based on bioinformatics data, it is suggested that Arctiin can exert its anti-necrotic and apoptotic effects by directly targeting RIPK1 and/or MLKL. Shizuka Koshinuma et al.’s experiments perfusing guinea pig hearts demonstrate that concurrent inhibition of necroptosis and apoptosis improves cardioprotection (177). Therefore, the therapy of combining RIPK inhibitors with standard statin presents an appealing option for preventing cardiovascular complications associated with diabetes. Additionally, RIPK inhibitors show the possibility in replacing aspirin for preventing cardiac malignancies, contingent on addressing the issue of hepatoxicity.

### 4.3 Role of RIPK1 and RIPK3 in diabetic nephropathy

Diabetic nephropathy (DN) is a frequent and severe complication of DM, marked by the reduction of GFR and the elevation of urinary protein excretion. As DN advances, it may result in serious complications like renal insufficiency, hypertension, and cardiovascular disease, ultimately leading to heightened all-cause mortality risk in patients. Additionally, common symptoms of DN like edema, anemia, and fatigue significantly impact patients’ daily lives. The disease’s progression may require long-term dialysis or kidney transplantation, resulting in repeated treatments, extended care, hefty financial burden, and both physical and mental damage to patients.

Chronic inflammation in the kidney is a crucial pathological mechanism that triggers DN. Although there is ongoing debate regarding whether DN can be classified as an inflammatory condition, mounting evidence suggests that persistent inflammation of both circulatory system and renal tissue is a significant physiological component of microangiopathy in DN (178). In the prediabetic stage, it has been observed that there is a significant increase in glomerular filtration rate along with increased microproteinuria and C-reactive protein levels, which indicates that the inflammation has appeared at this stage. The work by Bohle’s team and subsequent research have confirmed macrophage infiltration in the renal tissues of diabetic patients. A considerable number of recent studies further reveal the role of inflammation and immune response in the development of DN.

The inflammation of DN is inherently linked with NF-κB, a crucial and pervasive transcription factor that can be rapidly activated by numerous inflammatory mediators found in DN (179). including hyperglycemia, advanced glycation end products (AGE), mechanical stress, ROS, inflammatory cytokines, angiotensin II (Ang-II), and albuminuria (180). activated NF-κB stimulates the transcription of pro-inflammatory cytokines, chemokines, and adhesion molecules (181). The development of drugs targeting the NF-κB pathway encounters many difficulties. This is primarily due to the pathway’s complexity, which makes drugs capable of easily causing harmful effects on other cells or tissues, thereby posing greater risks and side effects. Nevertheless, RIPK inhibitors, with their larger therapeutic window and fewer side effects, exhibit potential for development. Man Guo et al. created an *in vitro* cellular model to investigate the intervention of high glucose levels by using normal rat renal tubular cells (NRK-52E). Additionally, they utilized an *in vivo* mouse model of DN and found that Nec-1 and N-acetylcysteine (NAC) could improve renal function by inhibiting RIPK1 (182). Therefore, RIPK1 and targeted antioxidants may be a potential therapeutic target for DN.

This conclusion is also corroborated by alternative studies that identify necrotic apoptosis as a strongly inflammatory type of cell death, which plays a significant role in the advancement of DN (183). Yuyin Xu found that necroptosis might significantly contribute to foot cell injury and depletion in diabetic neuropathy by activating the RIPK1-RIPK3-MLKL signaling pathway. This was observed through measuring necroptosis, apoptosis, and apoptosis in foot cells *in vivo* and *in vitro (* 184). Xian Wang and colleagues’ research on Paeoniflorin (PF) demonstrates that PF directly binds and facilitates degradation of TNFR1 in podocytes (185), which, in turn, prevents injury to foot cells in DN by regulating necrotic apoptosis through the RIPK1-RIPK3 signaling pathway.

Many recent studies have reported that RIPK3 can independently promote cytokine release and the formation of inflammasome containing NLRP3 (186–188). Upon stimulation by danger-associated molecular patterns, the Pyrin structural domain of NLRP3 binds to apoptosis-associated speck-like proteins that contain cysteine-rich domain recruitment structural domains. These domains then bind to pro-caspase-1 via CARD-CARD interactions, converting pro-caspase-1 to active caspase-1. Meanwhile, pro-IL-1β is cleaved by inflammasome, resulting in the maturation of IL-1β and subsequent inflammatory responses (189, 190). Thus, it is conceivable that RIPK3 could function as an innovative target for inflammatory therapy in DN. Sadly, there are no on-going initiatives for developing inhibitors that target RIPK3 precisely, which exhibits potential as a research hotspot for future immune drug and inflammatory therapy development.

Similarly, there are reports on the role of RIPK in necrotic apoptosis, but few studies have directly explored the role of RIPK1 and RIPK3 in DN, and the relevant areas remain highly investigable. Therefore, further research is necessary to investigate the precise role of RIPK1 and RIPK3 in DN. Most known studies utilize acute injury models with increased necrotic apoptosis. For instance, the RIPK1 inhibitor Nec-1 can reduce renal ischemia and reperfusion injury as well as sepsis-related acute kidney injury (191, 192). Lack of RIPK3 can protect against renal tubular injury in acute kidney injury induced by sepsis (193, 194). Deletion of RIPK3 or MLKL can hinder oxalate crystal-induced acute kidney injury and renal damage in a mouse model of renal ischemia-reperfusion injury, among other effects (195–197). However, further experiments with chronic injury models are necessary to provide ample evidence supporting the role of RIPK inhibitors for DN. Nonetheless, given the inextricable link between necrotic apoptosis and the onset of inflammation, it is reasonable to hypothesize that inhibitors of RIPK1 and RIPK3 could aid in controlling DN and may prove more effective when used in conjunction with antihyperglycemic agents.

## 5 Conclusion

With the surge in the number of people suffering from the disease, diabetes mellitus has become a major challenge in the field of medicine and health. The current treatment model of type 2 diabetes mellitus is mainly based on lowering blood glucose, such as interfering with intestinal glucose absorption and increasing glucose utilization conversion in peripheral tissues, etc (198, 199). However, the incidence of chronic complications of T2DM is still high, and the effective effects of traditional drugs and the huge side effects of new drugs have also been a difficult problem for clinical application, so there is an urgent need of a new target drug based on the molecular mechanisms of diabetic inflammation in an attempt to radically reduce the harm of diabetic complications.

The pathological mechanism of diabetic complications cannot be separated from inflammation, and the extensive roles of RIPK1 and RIPK3 in NF-κB, necro-apoptotic pro-inflammatory pathways, and inflammatory crosstalk demonstrate the potential of targeting RIPK1 and RIPK3 as a therapeutic for diabetic complications. With the current enthusiasm of major pharmaceutical companies for RIPK1 inhibitors, there is an urgent need to develop inflammation-targeting agents for DM complications as soon as possible, based on existed clinical use.

However, it is important to point out that despite the anti-inflammatory effects of RIPK inhibitors, the problems of RIPK1 and RIPK3 being dependent on cell type has arisen in previous experiments (198, 200–202). Therefore, further development and refinement of the studies on human primary cells (especially human cells important for inflammation) is necessary, and we need to conduct further studies on the effects of RIPK inhibitors in general models of inflammation as well. Diabetic complications are not confined to a single site, but often involve the whole body, and more detailed studies on different cell types will further reveal the complex interactions of these small molecule antagonists in the inflammatory pathways associated with diabetic complications, including TNFR1, MAPK and NF-κB signaling. In addition, whether the role of drug development falls on the kinase binding process of RIPK1 and RIPK3 or the scaffolding structure of RIPK1 and RIPK3 is also worth discussing.

RIPKs form a convoluted and interconnected network of signaling pathways. However, RIPK1 and RIPK3 have distinct and interdependent inflammatory roles, thereby necessitating an investigation into both pathways when signaling from small molecules or small interfering RNA to identify specific scenarios where targeting either or both pathways could be advantageous. Are these two inflammatory pathways of disparate significance in distinct inflammatory scenarios? Do these pathways undergo dissimilar alterations in diverse anatomical structures and cell types, and what is the ideal dosage to attain an optimal outcome? Furthermore, which pathway plays the leading role in influencing biological outcomes? Do these pathways undergo dissimilar alterations in diverse anatomical structures and cell types, and what is the ideal dosage to attain an optimal outcome?

In conclusion, RIPK1/3 seems to be promising anti-inflammatory targets for diabetic complications. Although many RIPK1 inhibitors remain in early clinical phases, their toxicity and off-target issues impede development, likely due to drug absorption in the liver and RIPK1’s regulation of hepatocytes as a housekeeping factor for homeostasis and inflammation (203). For this reason, RIPK1 inhibitors are prone to hepatotoxicity. Learning from the experience of terminated RIPK1 inhibitors, the key challenges for future clinical development include tracking real-time activation or necrosis biomarkers of RIPK1 *in vivo* and developing highly specific, efficient, and safe small-molecule inhibitors of RIPK1. It is hypothesized that RIPK3 signaling inhibitors may be more suitable for specifically targeting inflammation-related signals, which requires further demonstration through relevant studies for drug development. It is hoped that these inhibitors will undergo testing in patients with T2DM complications and be implemented in clinical practices in the near future.

## Author contributions

DK: Conceptualization, Writing – original draft. ZZ: Funding acquisition, Writing – review & editing. JL: Funding acquisition, Writing – review & editing. PC: Writing – review & editing. YD: Writing – review & editing. XS: Writing – review & editing. YC: Funding acquisition, Supervision, Writing – review & editing. LL: Conceptualization, Funding acquisition, Supervision, Visualization, Writing – original draft.
